# Clinicians' perceptions of prescribing antibiotics for infections in the diabetic foot

**DOI:** 10.1186/1757-1146-8-S1-A5

**Published:** 2015-04-20

**Authors:** Debbie Sharman

**Affiliations:** 1 Dorset HealthCare University Foundation Trust, Dorset, UK

## Introduction

Responsible use of antibiotics is high on the public health agenda. The management of infection within the diabetic foot is variable, partly due to a lack of consensus and evidence to guide management choices. Study aims: To explore and describe experiences and views regarding the clinical management of diabetic foot infections among specialist secondary care clinicians involved in the management of diabetic foot infections; to explore perceptions and awareness of antibiotic resistance; to explore whether there are any unknown factors that may contribute to the ‘non-pharmacological’ prescribing of antibiotics. Study design and methods: A phenomenological approach was used, with data collected in face to face interviews with specialist hospital clinicians including Consultant Physicians, Specialist Registrars, Consultant Microbiologist, Specialist Podiatrists and Consultant Vascular Surgeons within two acute hospital foundation trusts in the south of England.

## Results

A total of 152 themes were identified and clustered into ten different groups including: resistance issues, appropriate use of antibiotics, when to prescribe / stop antibiotics, influences on prescribing, complications / areas of concern, microbiology, duration of treatment, the multi-disciplinary team (MDT), training / experience and other factors. There was generally a strong consensus of opinion among clinicians, despite the lack of availability of strong evidence to guide practice.

## Conclusion

Responsible use of antibiotics is high on the public health agenda. This study presents a new way to understand how specialist clinicians perceive antibiotic prescribing in the management of diabetic foot infection. Clinicians report the value of the multi-disciplinary foot care team and the importance of shared decision making. There is acknowledgement that antibiotics may be prescribed for longer durations than may be absolutely indicated, but a belief that current practices do not lead to excessive harm in terms of antibiotic resistance and significant adverse effects.

Antibiotics do not appear to be used indiscriminately, and careful consideration is given to their use. There is consensus regarding the limited value of superficial wound swabs, and a desire for improved diagnostic measures to guide treatment choices. Experience and clinical judgement are seen as the major tools available in terms of managing diabetic infection.

